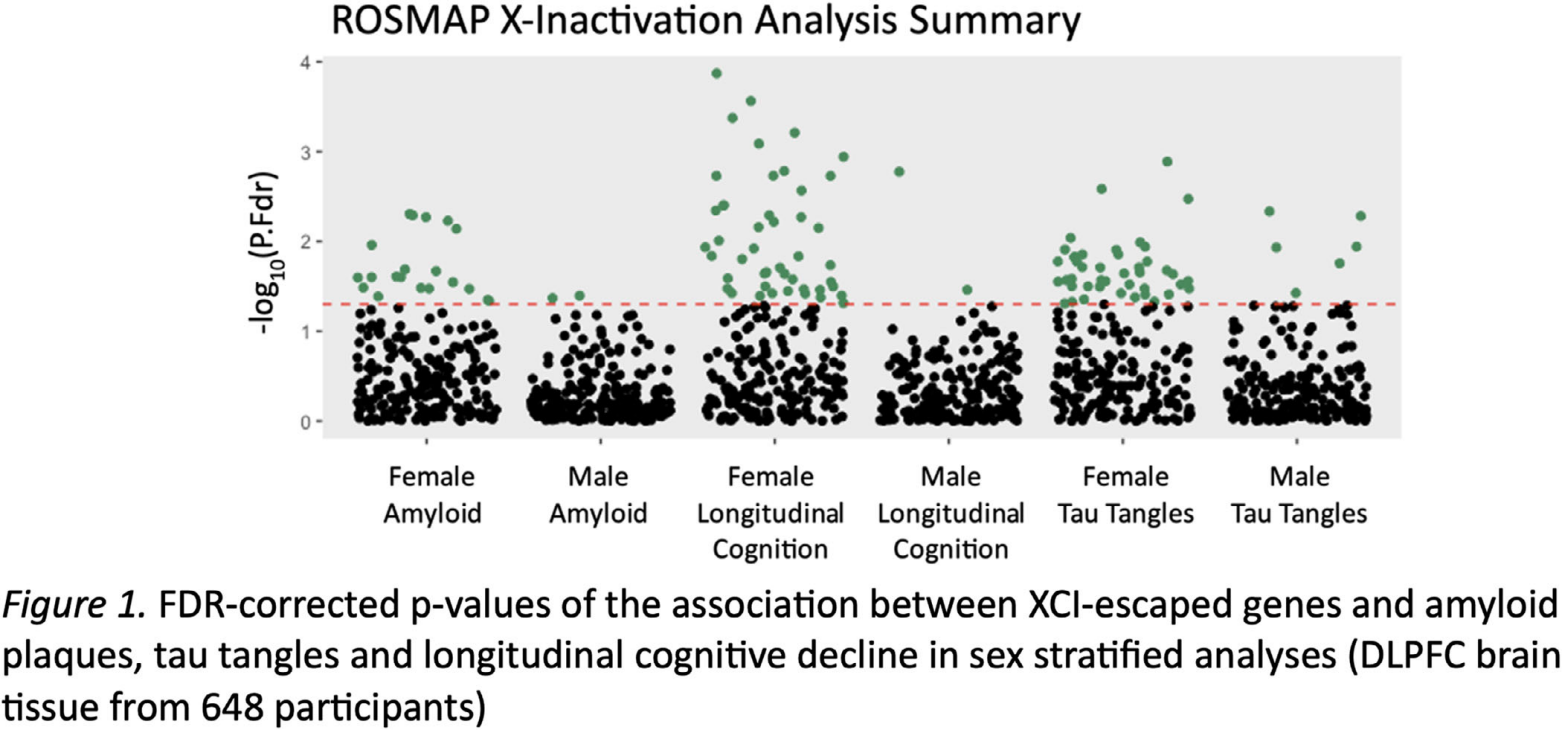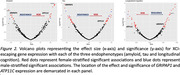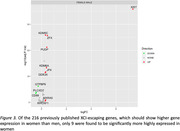# Genes that escape X chromosome inactivation are associated with Alzheimer’s disease endophenotypes: findings from ROSMAP

**DOI:** 10.1002/alz.090796

**Published:** 2025-01-03

**Authors:** Mabel Seto, Michelle Clifton, Gillian T Coughlan, Rory Boyle, Colin Birkenbihl, Ting‐Chen Wang, Philip L. De Jager, Julie A. Schneider, Reisa A Sperling, Yanling Wang, David A. Bennett, Timothy J. Hohman, Hyun‐Sik Yang, Logan C. Dumitrescu, Rachel F Buckley

**Affiliations:** ^1^ Center for Alzheimer’s Research and Treatment, Department of Neurology, Brigham and Women’s Hospital, Harvard Medical School, Boston, MA USA; ^2^ Vanderbilt Memory & Alzheimer’s Center, Vanderbilt University Medical Center, Nashville, TN USA; ^3^ Massachusetts General Hospital, Harvard Medical School, Boston, MA USA; ^4^ Harvard Medical School, Massachusetts General Hospital, Boston, MA USA; ^5^ Massachusetts General Hospital/Harvard Medical School, Boston, MA USA; ^6^ Vanderbilt Genetics Institute, Vanderbilt University Medical Center, Nashville, TN USA; ^7^ Columbia University Irving Medical Center, New York, NY USA; ^8^ Rush Alzheimer’s Disease Center, Rush University Medical Center, Chicago, IL USA; ^9^ Center for Alzheimer’s Research and Treatment, Brigham and Women’s Hospital, Massachusetts General Hospital, Harvard Medical School, Boston, MA USA; ^10^ Rush Alzheimer's Disease Center, Rush University Medical Center, Chicago, IL USA; ^11^ Vanderbilt Memory and Alzheimer’s Center, Vanderbilt University Medical Center, Nashville, TN USA

## Abstract

**Background:**

Women are disproportionately affected by Alzheimer’s disease (AD) and exhibit greater AD neuropathology than men. Women possess two X chromosomes, with one randomly silenced across each cell for dosage compensation. X chromosome inactivation (XCI) is not complete, and XCI‐escaping genes provide a promising avenue of discovery for biological pathways driving sex‐specific AD risk. Our objective was to examine XCI‐escaping genes in association with β‐amyloid (Aβ) and tau tangle density, and cognitive decline.

**Methods:**

Using bulk RNAseq from dorsolateral prefrontal cortex tissue, Aβ plaque and tau tangle pathology, and antemortem longitudinal cognition data from ROSMAP, we investigated whether XCI‐escaping genes explain significant variance in AD endophenotypes. Propensity scoring based on age‐at‐death, postmortem interval, race, latency‐to‐death, education, and *APOE*ε4 status resulted in a matched sample (N = 648, age‐at‐death_mean(SD)_ = 87.5(6.5)). Linear regression and mixed‐effects models assessed the association between 216 reported XCI‐escaping genes and Aβ and tau at autopsy, and a longitudinal global cognitive composite. Analyses were sex‐stratified and FDR‐corrected. Differential expression analyses assessed sex‐biased mean gene expression.

**Results:**

22 XCI‐escaping genes were associated with Aβ (20 female‐specific, 2 male‐specific), 49 genes with tau (43 female‐specific, 6 male‐specific), and 48 genes with cognitive decline (46 female‐specific, 2 male‐specific). In women, 40%(8/20) were negatively associated with Aβ, 56%(24/43) negatively associated with tauopathy, and 43%(20/46) were negatively associated with cognitive decline. Of note, higher *GRIPAP1* expression was associated with lower Aβ (β = ‐0.18, p_FDR_ = 0.02) and tau (β = ‐0.21, p_FDR_ = 0.001), and slower cognitive decline (β = 0.02, p_FDR_ = 0.04) in women. By contrast, *ATP11C* expression was associated with higher Aβ (β = 0.19, p_FDR_ = 0.03) and tau (β = 0.15, p_FDR_ = 0.03), and faster cognitive decline (β = ‐0.02, p_FDR_ = 0.03) in women. Unexpectedly, of 216 XCI‐escaping genes tested, only 4% were expressed more highly in females than males.

**Conclusion:**

*GRIPAP1* and *ATP11C* are implicated in endosomal recycling and inflammation, respectively, supporting two pathways associated with AD. Both *GRIPAP1* and *ATP11C* exhibited sex‐parity in gene expression suggesting that single‐cell RNAseq will be necessary to further characterize XCI‐escapism in relation to AD risk. Altogether, this study presents evidence that studying the X chromosome is integral to understanding female resistance, resilience, and vulnerability to AD pathology.